# Placental *FKBP5* Genetic and Epigenetic Variation Is Associated with Infant Neurobehavioral Outcomes in the RICHS Cohort

**DOI:** 10.1371/journal.pone.0104913

**Published:** 2014-08-12

**Authors:** Alison G. Paquette, Barry M. Lester, Devin C. Koestler, Corina Lesseur, David A. Armstrong, Carmen J. Marsit

**Affiliations:** 1 Department of Pharmacology and Toxicology, Geisel School of Medicine at Dartmouth, Hanover, New Hampshire, United States of America; 2 Department of Pediatrics, Center for the Study of Children at Risk, Women and Infants Hospital, Warren Alpert Medical School of Brown University, Providence, Rhode Island, United States of America; 3 Department of Biostatistics, University of Kansas Medical Center, Kansas City, Kansas, United States of America; 4 Department of Community and Family Medicine, Section of Biostatistics and Epidemiology, Geisel School of Medicine at Dartmouth, Hanover, New Hampshire, United States of America; University of Iowa Hospitals & Clinics, United States of America

## Abstract

Adverse maternal environments can lead to increased fetal exposure to maternal cortisol, which can cause infant neurobehavioral deficits. The placenta regulates fetal cortisol exposure and response, and placental DNA methylation can influence this function. FK506 binding protein (FKBP5) is a negative regulator of cortisol response, *FKBP5* methylation has been linked to brain morphology and mental disorder risk, and genetic variation of *FKBP5* was associated with post-traumatic stress disorder in adults. We hypothesized that placental *FKBP5* methylation and genetic variation contribute to gene expression control, and are associated with infant neurodevelopmental outcomes assessed using the Neonatal Intensive Care Unit (NICU) Network Neurobehavioral Scales (NNNS). In 509 infants enrolled in the Rhode Island Child Health Study, placental *FKBP5* methylation was measured at intron 7 using quantitative bisulfite pyrosequencing. Placental *FKBP5* mRNA was measured in a subset of 61 infants by quantitative PCR, and the SNP rs1360780 was genotyped using a quantitative allelic discrimination assay. Relationships between methylation, expression and NNNS scores were examined using linear models adjusted for confounding variables, then logistic models were created to determine the influence of methylation on membership in high risk groups of infants. *FKBP5* methylation was negatively associated with expression (*P* = 0.08, r = −0.22); infants with the TT genotype had higher expression than individuals with CC and CT genotypes (*P* = 0.06), and those with CC genotype displayed a negative relationship between methylation and expression (*P* = 0.06, r = −0.43). Infants in the highest quartile of *FKBP5* methylation had increased risk of NNNS high arousal compared to infants in the lowest quartile (OR 2.22, CI 1.07–4.61). TT genotype infants had increased odds of high NNNS stress abstinence (OR 1.98, CI 0.92–4.26). Placental *FKBP5* methylation reduces expression in a genotype specific fashion, and genetic variation supersedes this effect. These genetic and epigenetic differences in expression may alter the placenta’s ability to modulate cortisol response and exposure, leading to altered neurobehavioral outcomes.

## Introduction

The developmental origins of health and disease model has highlighted the significance of the fetal environment on neurological and behavioral outcomes [1]. The fetus can be exposed to maternal cortisol in circulation, and excessive cortisol exposure, associated with maternal adversity, is also associated with neurological and developmental problems [2]. Prenatal cortisol exposure predicts infant cortisol response to acute stress [3], and cortisol reactivity is associated with infant behavioral reactivity [4]. In humans, pregnancy stress is associated with increased incidence of affective disorders, including attention deficient disorder [5], [6], anxiety, and lowered cognitive and language abilities [7]. These associations are likely mediated by altered fetal cortisol exposure and reactivity, but the molecular mechanisms underlying this relationship remain unclear.

The placenta, a central regulator of fetal development, is a plastic organ that can respond to maternal signals and contribute to developmental outcomes by altering the environment of the developing fetus [8]. Alterations to placental function can occur through epigenetic mechanisms, including DNA methylation, which in regulatory regions alters gene transcription or transcription potential [9]. Placental methylation patterns influence global placental gene expression, and thus function, during crucial developmental periods [10]. Patterns of placental DNA methylation are dynamic, can be modified by various factors in the maternal environment [11]–[15], and can influence infant neurological, behavioral and cognitive outcomes. Methylation of cortisol response genes 11-beta hydroxysteroid dehydrogenase type 2 (*HSD11B2*) and the glucocorticoid receptor (*NR3C1)* have been associated with infant clinical and neurobehavioral outcomes, quantified through the Neonatal Intensive Care Unit (NICU) Network Neurobehavioral Scales (NNNS), a validated assessment to quantify newborn and infant neurobehavior [11], [16], [17].

*FKBP5 (*FK506 binding protein*)* decreases the binding of cortisol to its receptor and impedes nuclear translocation [18], leading to reduced cortisol response. *FKBP5* methylation within a regulatory region of intron 7 has been associated with neurological outcomes [19], [20]. Intron 7 has been shown to bind to the transcriptional start site of *FKBP5* using chromatin confirmation capture, and methylation alters *FKBP5* induction [20]. Glucocorticoid exposure has been associated with alterations to *FKBP5* intronic methylation in the hippocampus and blood of mice [21]. In a human hippocampal progenitor cell line, cortisol induced de-methylation of this regulatory region within intron 7 during critical periods of cellular differentiation and proliferation [20], which may be particularly relevant during fetal development, when cells are rapidly proliferating. *FKBP5* polymorphisms are also associated with altered induction of mRNA, changes to hippocampal size [20], [22], as well as diseases associated with alterations in the hypothalamic-pituitary-adrenal (HPA) axis, such as post-traumatic stress disorder (PTSD) [23]–[27]. The SNP rs1360780 is located near a functional response element within intron 2, and is strongly associated with diseases involving dysregulation of the HPA axis [28]–[30]. *FKBP5* expression is induced in a genotype specific manner, as the T allele binds to the transcriptional start site to enhance transcription [31]. The CT and TT genotype are associated with increased cortisol activity and altered physiological stress regulation in infants [32]. Epidemiological evidence suggests that a combination of genetic polymorphisms and cortisol induced *FKBP5* DNA methylation are associated with HPA axis related disorders.

FKBP5 is expressed within the placenta [33], but its functional relevance and potential for epigenetic control remain unknown. Since the placenta regulates fetal exposure and response to maternal cortisol, placental FKBP5 could potentially alter fetal development, particularly neurobehavioral development. The aims of this study were to determine the relationship between placental *FKBP5* methylation, genetic variation, and placental gene expression, and to determine if differential methylation and genotype associated with infant neurodevelopmental outcomes related to dysregulation of the HPA axis. In this study, we show that *FKBP5* intron 7 methylation reduces placental gene expression, genetic variation at rs1360780 influences placental gene expression, higher *FKBP5* intron 7 methylation is associated with increased risk of high infant arousal, and the TT genotype of rs1360780 is associated with high infant stress abstinence as quantified using the NNNS, a series of validated, quantitative measures of infant neurobehavior.

## Results

Demographic characteristics of the 509 infants in this study are shown in *Table 1*. All infants were >37 weeks gestation, with a mean gestational age of 39.01 weeks. There was a nearly identical proportion of females compared to males (50.7% vs. 49.3%). This study oversampled for large for gestational age (LGA, >90th BW percentile, 25.9%) and small for gestational age (SGA, <10th BW percentile, 20.0%) infants. All infants were genotyped for the SNP rs1360780, and the minor genotype frequency (TT) was 0.07. The distribution of genotypes is in Hardy-Weinberg equilibrium, and is similar to the expected frequency within the CEU population according to dbSNP.

**Table 1:** Infant Clinical and Demographic Information and relation to *FKBP5* methylation.

	N	%	Mean	Std Dev.	Mean *FKBP5* Methylation	P
**Maternal age**	509		29.32	5.53		
**Pregnancy tobacco use**						**0.35**
Yes	22	4.3			87.89	
No	481	94.5			87.12	
NA	6	1.2				
**Birth weight grams)**	**509**		**3489**	**682.54**		**0.01**
**Gestational age (weeks)**	509		39.01	0.95		0.32
**Birth weight Category**						**0.03**
LGA	132	25.9			86.74	
AGA	275	54			87.06	
SGA	102	20			87.9	
**Gender**						**0.03**
Female	258	50.7			86.82	
Male	251	49.3			87.47	
**rs1360780**						**0.09**
CC	231	45.4			86.9	
CT	235	46.2			87.11	
TT	35	6.9			88.25	
NA	8	1.6				

We quantified methylation of 2 CpGs through pyrosequencing of placenta samples in 509 infants, and methylation of these 2 CpGs was well correlated (Cor = 0.35, P = 4.44×10^−16^) and and as expected each was correlated to the mean of the 2 sites (Correlation of CpG 1 with mean: cor = 0.77, P = 2.2×10^−16^; correlation of CpG 2 with mean: cor = 0.86, P = 2.2×10^−16^). Thus, to reduce multiple comparisons, the mean methylation of these sites was used for further analysis. In this population, the mean methylation of the CpGs examined was 87.15% (range 76.15%–98.82%, SD 3.44%), and this data was normally distributed. The relationship between methylation and infant clinical outcomes was examined and shown in *Table 1*. There were subtle, statistically significant differences in methylation based on infant birth weight category, and infant gender. We observed that SGA infants had slightly higher methylation (87.9%) than AGA and LGA infants (87.06% and 86.74%, respectively) (*P* = 0.03, ANOVA). Males had higher methylation than females (87.47% compared to 86.82%, *P* = 0.03). Infants with the CC and CT genotypes had lower methylation than the TT genotype (CC and CT mean = 87.01, TT mean = 88.25, *P* = 0.03, T-Test), although this difference was attenuated when individuals are stratified by individual genotype (*P* = 0.09, ANOVA).

**Figure 1:**
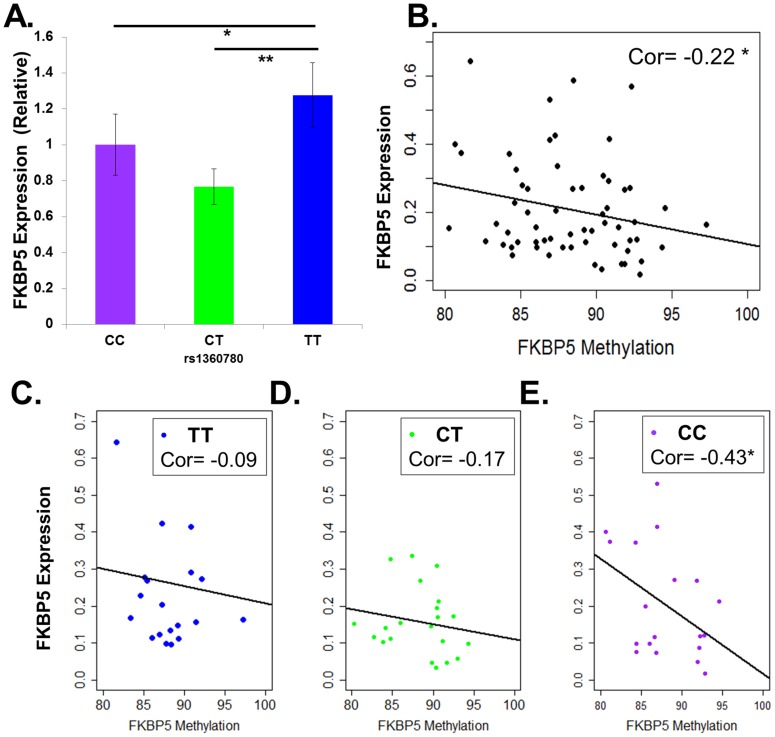
Methylation and Genotype are associated with FKBP5 Expression. Gene expression results were quantified in a subset (N = 61) of all placentas sequenced **A.**Gene expression as quantified as *FKBP5*/*SDHA* counts stratified by genotype, divided by the mean value of the average of the CC group (*P* = 0.07, ANOVA, Tukey test CT vs. CC, *P* = 0.53. CC vs TT *P* = 0.41, TT vs CT *P* = 0.05) **B.** Correlation of *FKBP5/SDHA* counts vs. *FKBP5* Intron 7 Methylation (r = −0.22, *P* = 0.08**). C–E.** Correlation of *FKBP5/SHDA* counts vs. *FKBP5* Intron 7 methylation stratified by genotype.*P≤0.1 ** = P≤0.05.

*FKBP5* gene expression was quantified in a subset of 63 placentas, 21 randomly selected from each genotype. After assessing ddPCR results for quality control, the N was reduced to 61 (19 CC, 21 CT, 21 TT genotypes). Gene expression was calculated as the counts of the *FKBP5* divided by the counts of *SDHA* as a housekeeping gene. To determine the association between expression and genotype, mean methylation of CC was set as the reference genotype, and with the mean of CT and TT genotypes normalized to this value. In Figure 1 A, comparing across the three genotypes suggested differences in expression by genotype (P = 0.07 ANOVA), with statistically significant differences between the TT and CT genotypes (P = 0.05, Tukey’s test), and no differences between the CT and CC genotype (P = 0.43, Tukey’s test). Among all genotypes combined, gene expression was negatively correlated with methylation, although this did not attain statistical significance at the *P* = 0.05 level. (Cor = −0.22, *P* = 0.08, Figure 1B). Based on previous evidence of genotype specific associations between methylation and expression [20], we stratified by rs1360780 genotype (Figure1 C–E), which revealed that the C allele appeared to be driving this relationship. The CC genotype showed a strong correlation between methylation and expression that approached but did not obtain statistical significance at the *P* = 0.05 level (Cor = −0.43, *P* = 0.06, Figure 1C), whereas there was no correlation between methylation and expression observed with the CT genotype (Cor = −0.17, *P* = 0.46, Figure 1D), or with the TT genotype (Cor = −0.10, *P* = 0.67 Figure 1E).

We fit a series of 6 linear regression models to determine the role of *FKBP5* methylation and the rs1360780 genotype on infant neurobehavioral outcomes. We focused on the 6 infant NNNS outcomes (habituation, attention, stress-abstinence, quality of movement, handling, and arousal) found to be most sensitive in prior studies [34], [35]. Each of the models was adjusted for infant birth weight group, maternal age, sex, and rs1360780 genotype, with CC as the referent genotype (Table 2). We observed a significant positive relationship between *FKBP5* methylation and infant arousal in the unadjusted model (Estimate = 0.02, *P* = 0.03), which was strengthened in the adjusted model (Estimate = 0.03, *P* = 0.005). Infants with the TT genotype of rs1360780 had increased stress abstinence scores compared to infants in the CC genotype (Estimate = 0.03, *P* = 0.03), and this relationship was maintained in the adjusted model (Estimate = 0.02, *P* = 0.04). Infants with the CT genotype did not display increased stress abstinence compared to infants with the CC genotype in the adjusted or unadjusted models.

**Table 2:** Linear regression model of *FKBP5* methylation, rs1360780 Genotype and NNNS outcomes.

	Unadjusted			Adjusted*		
	Estimate	Std.Error	*P*	Estimate	Std.Error	*P*
***Habituation***						
Methylation	−0.03	0.02	0.21	−0.04	0.02	0.12
CC	*ref.*			*ref.*		
CT	0.24	0.17	0.15	0.23	0.17	0.18
TT	0.55	0.33	0.10	0.64	0.33	0.05
***Attention***						
Methylation	0.01	0.02	0.43	0.01	0.02	0.44
CC	*ref.*			*ref.*		
CT	0.06	0.13	0.64	0.04	0.13	0.78
TT	0.34	0.25	0.18	0.37	0.26	0.16
***Handling***						
Methylation	0.00	0.00	0.55	0.00	0.00	0.53
CC	*ref.*			*ref.*		
CT	0.03	0.02	0.16	0.03	0.02	0.15
TT	0.04	0.04	0.41	0.03	0.04	0.45
***Quality of movement***						
Methylation	0.00	0.01	0.73	0.00	0.01	0.60
CC	*ref.*			*ref.*		
CT	−0.01	0.06	0.81	−0.01	0.06	0.91
TT	−0.16	0.12	0.18	−0.15	0.12	0.21
***Arousal***						
Methylation	0.02	0.01	0.03	**0.03**	**0.01**	**0.005**
CC	*ref.*			*ref.*		
CT	−0.02	0.07	0.81	−0.01	0.07	0.84
TT	0.17	0.15	0.23	0.15	0.14	0.29
***Stress abstinence***						
Methylation	0.00	0.00	0.17	0.00	0.00	0.19
CC	*ref.*			*ref.*		
CT	0.00	0.01	0.83	0.00	0.01	0.75
TT	0.03	0.01	0.03	**0.02**	**0.01**	**0.04**

*Also adjusted for birth weight group, maternal age, gender and random effect of conversion plate.

To improve interpretability, infants were dichotomized into high vs. normal arousal and stress abstinence groups using normative scales by Fink et al [36]. Infants defined as high arousal (scores >5.14), represented 16.3% of our population (N = 83). Infants defined as high stress abstinence (scores >0.2) represented 38.3% of our cohort (N = 195). As we observed differential effects of methylation on expression by genotype, we sought to control for genotype in our models of methylation and NNNS outcomes. Thus we fit logistic regression models controlled for infant birth weight group, maternal age, sex, and rs1360780 genotype (*Table 3*). We stratified methylation into quartiles (Quartile 1: 76.15%–<85.06%, Quartile 2: 85.06%–<87.28%, Quartile 3: 87.28%–<89.23%, Quartile 4: 89.2%–98.82%), and analyzed associations with methylation as a categorical variable. Infants with methylation in the highest quartile demonstrated a 1.83 fold increased odds of being classified as high arousal (95% CI 0.91–3.68) compared to infants in the lowest quartile in an unadjusted model, and the risk became greater in the adjusted model (OR 2.22, 95% CI 1.07–4.61). Additionally, we observed that infant birth weight category was independently associated with infant arousal group, with SGA infants having a lower odds of high arousal in the adjusted model compared to AGA infants (OR 0.44, CI 0.20, 0.96), and LGA infants having a higher odds of high arousal than AGA infants (OR 1.76, CI 1.01, 3.04). Males had lower odds of high arousal compared to females (OR 0.52, CI 0.31, 0.85). We observed no interaction between gender and birth weight on the relationship observed between methylation and infant arousal (data not shown). Maternal age and genotype did not significantly influence membership in this group.

**Table 3:** Logistic regression model of *FKBP5* methylation quartiles and 95^th^ percentile arousal and stress abstinence scores.

	Arousal>5.14						Stress Abstinence>0.2				
	N (%) lowArousal		N (%) high Arousal	Unadjusted (OR, CI)		Adjusted (OR, CI)	N (%) low Stress Abstinence		N (%) high Stress Abstinence	Unadjusted (OR, CI)	Adjusted (OR, CI)
***Methylation Quartile***											
1^st^ Quartile	112 (88)	15 (12)		*Ref.*	*Ref.*			88 (69)	39 (31)	*Ref.*	*Ref.*
2^st^ Quartile	105 (83)	22 (17)		1.57(0.77, 3.19)	1.46 (0.71, 3.03)			74 (58)	53 (42)	1.63 (0.95, 2.79)	1.62(0.94,2.8)
3^st^ Quartile	106 (83)	21 (17)		1.48(0.72, 3.03)	1.52 (0.73,3.19)			73 (57)	54 (43)	1.41 (0.82, 2.44)	1.34 (0.77, 2.33)
4^st^ Quartile	103 (80)	25 (20)		1.83 (0.91, 3.68)	2.22(1.07,4.61)			79 (62)	49 (38)	1.23 (0.71,2.13)	1.23(0.7, 2.17)
***Birth weight Group***											
AGA	231 (84)	44 (16)		*Ref.*	*Ref.*			173 (63)	102 (37)	*Ref.*	*Ref.*
LGA	102 (77)	30 (23)		1.57(0.93, 2.64)	1.76 (1.01,3.04)			88 (67)	44 (33)	1.06 (0.67,1.69)	1.05 (0.65,1.69)
SGA	93 (91)	9 (9)		0.5 (0.23, 1.07)	0.44(0.2,0.96)			53 (52)	49 (48)	1.23 (0.76, 2.01)	1.17 (0.71, 1.94)
Maternal Age				1 (0.96, 1.04)	0.98 (0.94, 1.03)					0.98 (0.94,1.01)	0.97 (0.94,1.01)
***rs1360780***											
CC	193 (84)	38 (16)		*Ref.*	*Ref.*			149 (65)	82 (35)	*Ref.*	*Ref.*
CT	199 (85)	36 (15)		0.92 (0.56, 1.51)	0.93 (0.55, 1.55)			143 (61)	92 (39)	1.08(0.72, 1.62)	1.1 (0.73, 1.65)
TT	28 (80)	7 (20)		1.27 (0.52, 3.12)	1.2 (0.46, 3.11)			17 (49)	18 (51)	2.01(0.95, 4.26)	1.98(0.92,4.26)
Female	207 (80)	51 (20)		*Ref.*	*Ref.*			152 (59)	106 (41)	*Ref.*	*Ref.*
Male	219 (87)	32 (13)		0.59 (0.37, 0.96)	0.52 (0.31, 0.85)			162 (65)	89 (35)	0.81 (0.55, 1.18)	0.77 (0.52, 1.14)

There was no statistically significant association between high stress abstinence and *FKBP5* methylation quartiles (*see* *table 3*). Infants with the TT genotype had borderline significantly higher odds of being in the high stress abstinence group (OR 1.98, CI 0.92–4.26) compared to CC infants in the adjusted model. Infants with the CT genotype did not have statistically higher odds of increased stress abstinence compared to CC infants (OR 1.1, CI 0.73–1.65). Maternal age, infant birth weight group, and gender appeared to have no effect on membership in the high stress abstinence group in the adjusted model.

## Discussion

We analyzed placental methylation of the functionally relevant intron 7 of *FKBP5*, a negative regulator of cortisol response, and we observed that infants with higher methylation were at increased risk of exhibiting high arousal NNNS scores. We also found that infants with the minor genotype of rs1360780 were more likely to exhibit high stress abstinence independently of intron 7 methylation. The placenta modulates fetal exposure to maternal cortisol, and *FKBP5* is expressed within the placenta [33], but its role in the regulation of cortisol response within the placenta remains unknown. This study is the first to examine the consequences of genetic and epigenetic variation on placental gene expression and infant neurobehavioral outcomes.

Prior work has demonstrated that there is a complicated control of *FKBP5* expression based on interactions between various intragenic regions with the transcriptional start site (TSS) [20]. Chromatin confirmation capture uses a series of cross linking and restriction digests to identify three dimensional spatial relationships within genes; specifically where different parts of the DNA interact [37]. Chromatin conformation assays have revealed that intron 7 binds to the TSS, inducing transcription, and that DNA methylation within intron 7 may inhibit this binding, resulting in reduced expression [20]. Similarly, intron 2 interacts with the TSS in an allele specific manner, enhancing expression [20]. We have demonstrated higher placental expression of *FKBP5* from infants homozygous for the T allele compared to the heterozygotes and homozygous C infants. We also demonstrated that methylation of intron 7, as expected, was negatively correlated with expression, but only amongst those newborns with the CC genotype. The relationship between methylation and expression is not observed in individuals with the TT and CT genotype, suggesting that the T allele is also influencing *FKBP5* expression, resulting in increased expression of *FKBP5* and a loss of relationship between methylation and expression. These results are consistent with the model proposed by Klengel et al [20], suggesting that the effect of the T allele in intron 2 is dominant over the effect of methylation at intron 7.

In our study, infants with the highest quartile of methylation of *FKBP5* intron 7 were over twice as likely to have NNNS arousal scores in the highest 5^th^ percentile compared to infants in the lowest quartile of methylation. Newborn arousal is a behavioral measure reflective of the infant’s state and motor activity during the examination [38]; infants in the high arousal group would display increased fussiness, more spontaneous movements, and those with high arousal scores demonstrated higher risk scores in subsequent prenatal screening using the Hobel and Verma Scales [36]. Infants with the highest levels of methylation would experience reduced *FKBP5* expression, resulting in increased cortisol activation of glucocorticoid receptors targets within the placenta, which can influence activation of the glucorticoid response pathway in the developing infant. Previous studies of *FKBP5* have observed associations with post-traumatic stress disorders [24], [26], and PTSD is characterized by hypervigilance and hyperarousal. Thus, it appears that alterations to *FKBP5* and altered cortisol response influence behavior in a manner promoting a hypervigilant response. Individuals with higher *FKBP5* methylation in utero thus may have an over-active cortisol response pathway at the time of birth, and may be predisposed to develop disorders related to elevated HPA axis activity, such as post-traumatic stress disorder or anxiety.

We also observed a relationship between the rs1360780 genotype and risk of membership in the highest 5^th^ percentile of stress abstinence. The stress abstinence summary score is a measure of signs of physiological stress exhibited by the infant [38], and infants in the highest 5^th^ percentile as measured by Fink et al [38] showed signs of stress or abstinence on at least 11 of the 49 stress abstinence signs assessed. High levels of stress abstinence are generally associated with prenatal risk factors such as maternal tobacco use [39], drug exposure [38] or labor complications [36]. We found that infants that possessed the TT genotype had increased odds of membership in this group, and 51% of the infants classified as high stress abstinence possessed the TT genotype; a much higher proportion than the CC and CT genotypes. We note that this relationship did not reach statistical significance, likely due to the low prevalence of the TT genotype (6.9%). We showed that infants possessing the TT genotype have higher expression of *FKBP5* in the placenta, and we hypothesize that this may reduce the activation of other glucocorticoid response elements (such has HSD11B2), which may result in increased fetal exposure to maternal cortisol. More detailed analyses of the relationship of DNA methylation and expression across the family of genes involved in cortisol regulation is warranted.

We assume that infants with the TT genotype likely have higher expression in other tissues throughout gestation, including the brain, as the genotype would be constant in all tissues, and as we have shown, appears to supersede methylation in driving expression. Interestingly, the TT genotype has been associated with heightened cortisol reactivity in infants [32] as well as adults [28]. This effect is thought to reflect disruption of the negative feedback loop over time due to altered homeostatic balance of FKBP5 as a negative regulator, resulting in impaired recovery from stressful situations [32]. Such a model is consistent with our observed increased signs of stress abstinence, which suggest impaired recovery from the stresses of the prenatal period and/or delivery. Thus, these infants, who were born with elevated expression of *FKBP5* due to their genotype and already showed elevated stress abstinence at the time of birth, may also be predisposed to diseases associated with dysregulation of the HPA axis. More follow up work is needed to connect our observed associations of NNNS outcomes with later life psychological distress, as well as the interaction with other regulators of glucocorticoid response.

*FKBP5* placental methylation is not a single predictor of infant cortisol response, and we have a limited understanding of the contribution of this methylation with other factors that can influence infant neurobehavior; specifically infant gender and infant birth weight, as well as methylation of other genes involved in cortisol response, including *HSD11B* and *NR3C1* [11], [16], [17]. There are sexual dimorphisms in the onset and characteristics of many behavioral disorders, which may be attributed to differential DNA methylation in the placenta [40]. We observed that although male infants had very slightly higher *FKBP5* methylation than females, they were independently less likely to be classified as high arousal in our adjusted logistic model. Sex appears to have an influence on neurobehavioral outcomes that is independent of methylation within this population. The difference in methylation between sexes suggests that sex hormones or sex-differentiating factors may alter the mechanisms by which methylation patterns are set in the placenta at specific regions, and more work is needed to explore potential interactions between sex specific epigenetic patterning and neurobehavioral outcomes. Birth weight can broadly represent the quality of the intrauterine environment, and both LGA [41]and SGA [42] infants exhibit increased risk of neurological and cognitive deficits. We observed that although SGA infants had slightly higher methylation of the *FKBP5* intron 7 than LGA infants, they had decreased odds of being high arousal compared to AGA infants, while LGA infants were more likely to be classified as high arousal, controlled for methylation status. Both *FKBP5* placental methylation and birth weight appear to contribute independently to risk of high infant arousal, although *FKBP5* methylation is a stronger predictor than infant birth weight alone. We did not observe an interaction of these two factors, but a larger population may be necessary to observe such an interactive effect, especially owing to the very small magnitude of the association. This relationship would need to be explored in more detail in a population of infants with a normal distribution of birth weights.

The RICHS cohort represents a relatively healthy sample of infants born from non-pathologic, low-risk pregnancies, and therefore is not appropriate for examining certain stressors such as illicit drug use or maternal smoking. We would encourage the examination of this gene in appropriate studies that characterize the effects of these more extreme exposures and conditions to provide additional insight on the role of placental *FKBP5* in behavioral and mental disorder risk with more extreme stressors. Our work suggests that there may always be some level of epigenetic variability in *FKBP5* intron 7 present in placenta at the time of birth, and that this DNA methylation along with genetic variation have the potential to alter gene expression. The potential contributors to this variability in methylation remain unclear, but as RICHS represents a healthy newborn cohort from low risk pregnancies, it is most likely related to factors and exposures experienced by the population at large. This assessment has established predictive value, showing efficacy in predicting motor outcomes in older infants as quantified through the Bayley psychomotor developmental index [34] and NNNS scores have been shown to prospectively relate to medical and behavioral problems at 4 ½ years of age [35]. In addition, NNNS scores have been correlated with changes in brain physiology as quantified through MRI [43] [44].

This large study is the only one, to our knowledge, that has associated *FKBP5* placental methylation with neurobehavioral outcomes. We have observed associations between genetic variation and methylation, which both have been previously shown to contribute to cortisol reactivity [20]. We observed associations between these epigenetic changes and genetic variability with altered neurodevelopment, which is important because it reveals a potential novel role for *FKBP5* within the placenta. However, we are limited in the scope of which we can study the functional consequences of placental methylation. The placenta is a transient and dynamic tissue throughout development and is a relevant biomarker of the fetal environment, and tissue collected at term represents the end of this process. Placental gene expression and methylation change over the course of fetal development [10], thus the changes we observed may not be reflective of methylation in early development. *FKBP5* has been shown to be expressed within the placenta [33], but it is unclear from these results which specific cell type expresses *FKBP5* within the placental tissue. The results presented may be confounded by the heterogeneity of placental tissue we have gathered as the epigenetic changes that occur here may be occurring in a specific subset of placental cells. This, in fact, may explain the relatively small differences in methylation observed between groups. It may be possible to adjust for cellular proportions in more large scale methylation data, although this has only been done in limited data sets [45]. The correlation between methylation and expression in these infants is moderate (r = −0.22), but this level of correlation is expected as there is significant variability in the measurement of expression in the placenta, even given our relatively rigorous collection conditions. The type of and length of delivery time as well as other uncontrollable clinical factors could be affecting mRNA stability and thus expression, while DNA methylation is profoundly more stable, and is thus a more attractive biomarker for these studies.

Little is known of the function of FKBP5 within the placenta, and we can only make assumptions based on its role in other tissues. Animal studies have revealed that blood DNA methylation of *FKBP5* can be used as a surrogate marker of corticosteroid induced changes to *FKBP5* methylation in the hippocampus [21], although there have been no animal studies examining these correlations in placenta and brain, nor have these been demonstrated in human studies. We have previously observed that there is a great deal of variability in methylation by tissue and by gene within a tissue comparing infant cord blood, placenta and saliva, which are common biomarkers collected during early infancy [46], and so we would caution interpreting our results as directly reflecting the state of the hippocampus. However, we believe the placenta represents a functional tissue for cortisol regulation and can therefore influence neurodevelopmental programming. We encourage further analysis of the function of FKBP5 within the placenta to better understand this process, as well as work in model systems to understand the relationship between placental and brain *FKBP5*.

In our study design, we sought to observe associations between infant neurobehavior, methylation and expression, which involved testing multiple hypotheses and so there is a risk of Type I error, although we limited our analysis to the 6 most sensitive NNNS scores with the most predictive value. As the RICHS population represents newborn infants, little long term follow up data is available at the current time, and our analysis of infant behavior rests on the NNNS assessment, although the prospective validity of these assessments has been established [35], [47]. This assessment is highly robust and reliable measure of newborn neurobehavior and our assessment within the first few days of life removes the confounding variable of the postnatal environment.

Maternal cortisol has been shown to alter placental CRH, altering in utero cortisol exposure and the development of the fetal HPA axis through epigenetic mechanisms [48]. Cortisol has been shown to induce demethylation of *FKBP5* in neuronal progenitor cells [20], and we hypothesize that increased maternal cortisol may result in decreased *FKBP5* methylation, altering this stress response axis. We encourage further research (1) to determine the influence of maternal cortisol on fetal and placental *FKBP5* methylation, (2) to characterize how this methylation alters FKBP5 gene and protein expression and subsequent cortisol response over the course of gestation and (3) to validate our findings in additional cohorts and to expand this study in a population of more at risk infants. It would be useful to specifically test infant cortisol reactivity as a direct measure of physiologic programming. We provide an impetus for future work to study the physiology of FKBP5 protein in placental cortisol response and its potential long-term impact on child neurobehavioral development. Epigenetic regulation of *FKBP5* and other genes involved in placental cortisol response has been associated with a number of neurological outcomes individually, but the interactions between methylation patterning of these genes and their influence on cortisol response and neurobehavioral outcomes remains unclear. More sophisticated analysis of the contribution of cortisol response genes to placental cortisol response may further elucidate the contribution of placental epigenetic regulation to neurodevelopmental outcomes.

## Materials and Methods

### Study Population

The infants involved in this analysis represent all infants enrolled in the Rhode Island Child Health Study (RICHS) from September 2010 until February 2013 with neurodevelopmental assessments. RICHS recruits mother-infant pairs from Women and Infants Hospital of Rhode Island [17]. Infants considered LGA (large for gestational age) and SGA (small for gestational age) were selected and AGA (adequate for gestational age) infants matched on sex, gestational age (±3 days), and maternal age (±2 years) were also enrolled. Exclusion criteria included maternal life-threatening conditions or age <18 years and infant congenital or chromosomal abnormalities. Anthropometric and clinical data was collected from the inpatient medical record from delivery, and information about lifestyle, demographics, and exposure histories was obtained through an interviewer-administered questionnaire. Newborn neurobehavior was assessed via the NNNS, administered by certified psychometrists after the first 24 hours of life, but prior to discharge. Individual components of the NNNS were compiled into a series of 13 summary scores [49]. In this study, we focused on the summary scores (habituation, attention, stress-abstinence, quality of movement, handling and arousal) found to be most sensitive in prior studies [34], [35]. All patients provided written informed consent for participation under protocols approved by the institutional review boards at Women and Infants Hospital and Dartmouth College.

### Sample Collection, DNA extraction and bisulfite modification

For each subject, 12 samples of placental parenchyma (totaling approximately 10 grams of tissue) from standardized points 2 cm from the umbilical cord insertion site were excised from the fetal portion of the placenta, free of maternal decidua. Samples were placed immediately in RNAlater (Life Technologies) and stored at 4°C. At least 72 hours later, samples were removed from RNAlater, blotted dry, snap frozen in liquid nitrogen, pulverized to powder using a liquid nitrogen cooled stainless steel mortar and pestle (Cellcrusher), and stored at −80°C. DNA was extracted using the DNeasy Blood & Tissue Kit (Qiagen) and was quantified using the Nanodrop ND-2000 spectrophotometer (Thermo Fisher Scientific Inc.). 500 ng of DNA from each sample was bisulfite modified using the EZ-DNA methylation plate kit (Zymo Research) and stored at −20°C before analysis. All procedures were performed following manufacturer’s instructions.

### Bisulfite Pyrosequencing and DNA Methylation Analysis

All samples (N = 509) were sequenced for *FKBP5* intronic methylation. *FKBP5* intron 7 (GRCh37/hg19 Chr 6: 35,558,486–35,558,567) was amplified with a pyromark PCR kit (Qiagen) using primers (FKBP5-F, 5′- GGATTTGTAGTTGGGATAATAATTTGG -3′; and 5′–biotin- TCTTACCTCCAACACTACTACTAAAA -3″) and the following cycling conditions: 95°C for 9 minutes, followed by 42 cycles of 95°C for 30 seconds, 59°C for 1 minute and 72°C for 1 minute, and a final extension step of 5 minutes at 72°C. PCR products were sequenced using a PyroMark MD system (Qiagen), and the sequencing primer FKBP5 SEQ 5′- GGAGTTATAGTGTAGGTTT -3′. Each sequencing run contained no template and genomic DNA negative controls, as well as a methylated and unmethylated controls (Epitect). A bisulfite conversion control within the assay sequence was used to assess conversion efficiency.

### Genotyping

All samples (N = 509) were genotyped for the SNP rs1360780 through an allelic discrimination assay using predesigned Taqman primers (part #C___8852038_10, Life Technologies) and Taqman universal master mix (Life Technologies) using established protocols as directed by the manufacturer on a Bio-Rad CFX connect.

### Gene expression analysis

RNA was isolated from a subset of 21 randomly selected placentas from each genotype, for a total N of 63. Using the Qiagen RNeasy kit, RNA concentrations were quantified using ND-2000 spectrophotometer (Nanodrop), and RNA was stored at −80°C. For every sample, duplicate cDNAs as well as a non-template control were generated from 100 ng of mRNA using iScript cDNA master mix (BioRad) following manufacturer protocol and the resulting cDNA was stored at 4°C. mRNA was quantified using droplet digital PCR (ddPCR). Each cDNA was amplified in two technical replicates in different PCR reactions. Taqman gene expression arrays for the referent gene succinate dehydrogenase complex, subunit A (*SDHA*)[*50*] (part #Hs00188166_m1) and *FKBP5* (part #Hs01561004_m1, Life Technologies) were utilized. Droplets were generated using the QX100 droplet generator, amplified using standard conditions for the assays. Following amplification the droplets were quantified using the Biorad QX100 droplet reader using Quantasoft software (version 1.3.20). The relative amount of *FKBP5* was defined as the average *FKBP5* absolute counts divided by the average *SDHA* absolute counts from 4 replicates. The mean of the CC genotype was set as the reference and the expression data was divided by this mean to calculate the relative gene expression by genotype.

### Statistical Analysis

Two CpGs (Chr 6: 35558488 and 35558514) were sequenced based on a previous study [20]. Methylation was defined as the mean of these two CpG sites, and this data conformed to the normality assumption. Bivariate associations between methylation and NNNS scores were assessed with Pearson’s correlations. Student’s T-test or ANOVA were used to examine relationships between methylation and categorical variables, including genotype. Generalized linear mixed models (GLMMs) adjusted for birth weight group, maternal age, rs1360780 genotype, and a random effect variable for conversion plate to account for variations in conversion plate efficiency were created to explore the relationship between methylation, genotype, and 6 infant NNNS outcomes as continuous variables (habituation, attention, stress-abstinence, quality of movement, handling, and self-regulation) found to be most sensitive in prior studies [34] [35]. To improve interpretability, logistic GLMMS were used to model the relationship between methylation quartile and membership in the 95^th^ percentile of arousal and stress abstinence scores, based on the normative percentiles of Fink et al [36]. This model controlled for birth weight group, maternal age, rs1360780 genotype, and included a random effect variable for conversion plate to account for variations in conversion plate efficiency. GLMMs were fit using the R package NLME and LME4. All data was analyzed in R version 3.0.2[51].

## Supporting Information

Table S1 **Compiled methylation, genotype, and gene expression data with clinical covariates used for analysis, using de identified infant ID numbers.**
(XLSX)
